# Enhanced Nitrogen Dioxide Detection Using Resistive Graphene-Based Electronic Sensors Modified with Polymers of Intrinsic Microporosity

**DOI:** 10.1021/acssensors.4c03291

**Published:** 2025-02-17

**Authors:** Danielle M. Goodwin, Mariolino Carta, Muhammad Munem Ali, Daniel Gillard, Owen J. Guy

**Affiliations:** † Centre for Integrative Semiconductor Materials (CISM), Faculty of Science and Engineering, Swansea University—Bay Campus, Fabian Way, Swansea SA1 8EN, U.K.; ‡ Department of Chemistry, College of Science, Swansea University—Singleton Campus, Swansea SA2 8PP, U.K.

**Keywords:** graphene, gas sensors, surface modification, polymer of intrinsic microporosity (PIM), nitrogen dioxide

## Abstract

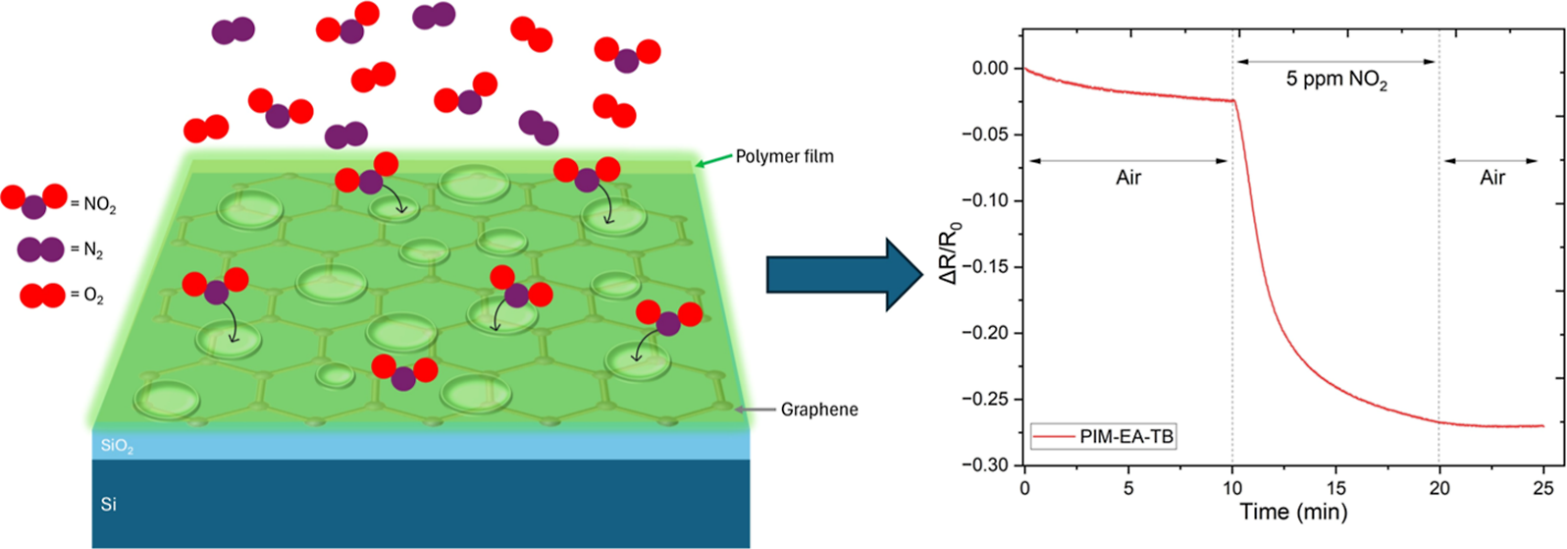

In this study, we report on the fabrication and evaluation of gas sensing performance for 3 × 3 graphene pixel array sensors coated with polymers of intrinsic microporosity (PIM-1 and PIM-EA-TB) and Matrimid, a commercial polyimide, for the detection of nitrogen dioxide (NO_2_). The polymer films, with thicknesses of only 9–11 nm, significantly enhanced the gas sensing performance, demonstrating responses as high as −25.7% compared to a bare graphene response of −10.8%. The gas sensing performance was evaluated in real-time by exposing the sensors to NO_2_ concentrations from 1 to 50 ppm, along with selectivity tests using ammonia (NH_3_), nitric oxide (NO), methane (CH_4_), and carbon dioxide (CO_2_). In addition to their high sensitivity, the sensors exhibited reduced response times by 56 s. They also demonstrated high selectivity for NO_2_, with minimal cross-sensitivity to other gases. Furthermore, the polymer membranes exhibited rapid recovery times (114–153 s) and limits of detection in the low parts per billion range, with PIM-EA-TB achieving a detection limit of 0.7 ppb. These features highlight their potential as promising candidates for real-time environmental monitoring of toxic gases, showcasing the potential use of PIMs to enhance the sensitivity and selectivity of graphene-based gas sensors and providing a foundation for further development of cost-effective and reliable NO_2_ detection systems.

Monitoring, controlling, and evaluating the health impact of air pollution has been a recent focus over the past decade.^1^ Nitrous oxides (NO_*x*_) are among the pollutants that can be found in ambient air and are associated with combustion. The World Health Organization (WHO) reports that concentrations of NO_*x*_ vary in different locations; however, they can exceed 500 μg/m^3^ (∼266 ppm) in busy urban areas.^2^ Exposure to high levels of nitrous oxides can damage respiratory organs; furthermore, low exposure can cause adverse effects such as shortness of breath and irritation to eyes, nose, throat, and lungs.^3^ On air quality standards, the European Commission reports that 40 μg/m^3^ is the maximum exposure limit averaging in 1 year that humans should be exposed to. Furthermore, given the WHO annual exposure limit for nitrogen dioxide (NO_2_) at just 21 ppb,^4^ the need for highly sensitive and selective detection methods for such gases is vital.

Graphene, an allotrope of carbon, is a two-dimensional (2D) single layer of sp^2^-hybridized carbon atoms arranged in a honeycomb structure.^5,6^ Its exceptional properties enable a wide range of applications, including in medicine,^7^ solar cells,^8^ energy storage,^9^ transparent electrodes,^10^ transistors,^11^ and nanocomposites.^12^ Graphene is also highly sensitive to various gases, including those found in the environment, as the adsorption of molecules on its surface can alter its carrier or electron mobility, resulting in measurable changes. Due to its unique physicochemical and biological properties, graphene has attracted significant attention for its potential in sensor technology.^13^ Although it has been integrated into various sensing platforms, challenges remain, including the scalable production of high-quality graphene,^14,15^ long recovery times or incomplete sensor recovery,^16^ and issues with its extreme sensitivity and poor selectivity, which can introduce noise and measurement variability. To address these limitations and improve sensor performance, surface modification techniques are typically employed to enhance both the sensitivity and selectivity. These modifications aim to tailor the graphene surface for specific target gases, enabling reproducible and reliable responses. One such technique involves adding a semipermeable membrane to the graphene surface,^16^ which can facilitate the selective and sensitive detection of specific gases. In one example, a poly(methyl methacrylate) (PMMA) membrane was added to a graphene-based sensor to enable selective filtration, improving its capability to detect hydrogen.^17^

Polymers of intrinsic microporosity (PIMs) are long chain, rigid polymers used in various applications including gas separation, membrane filtration, adsorption, and catalysis.^18,19^ This unique class of polymers features a structure with intermolecular voids, known as micropores (defined by IUPAC as pores smaller than 2 nm),^20^ which result from their rigid structure and limited rotational freedom. In fact, the rigid polymer backbone in PIMs primarily consists of aromatic monomers that possess “sites of contortion” that prohibit an easy conformational arrangement in the solid state. This lack of rotational freedom leads to the formation of micropores and internal free volume in the material.^21^ The combination of rigidity and porosity of PIMs, along with their solubility in common organic solvents, makes them highly suitable for applications in gas separation,^22^ exploiting their ability to selectively adsorb and transport gases based on molecular size and chemical properties. Leveraging the exceptional gas separation properties of PIMs for gas sensing could address some of the challenges associated with graphene’s extreme sensitivity and lack of selectivity. Despite their proven utility in gas separation,^23^ PIMs have not been extensively explored for gas sensing.^24^ The integration of a PIM membrane onto a graphene sensor could significantly enhance both the sensitivity and selectivity of the system, offering a promising approach for the development of more precise and reliable gas sensors.

In this study, we report the fabrication, surface modification, and evaluation of the gas sensing performance of graphene-based resistor sensors coated with two different PIMs, namely, PIM-1 and PIM-EA-TB as well as a commercial polymer, Matrimid, that was used for comparison. PIM-1, known as the “archetypal polymer of intrinsic microporosity”, has been extensively studied for several applications and is synthesized through the polycondensation of tetra-fluorophthalonitrile and a spirobisindane component (which provides the typical “site of contortion” necessary for a good PIM).^25^ PIM-EA-TB is a relatively new polymer synthesized using ethanoanthracene (EA) and Tröger base (TB) coupling.^26^ Matrimid, on the other hand, is a commercially available polyimide thermoplastic polymer widely used in gas separation applications^27,28^ and was utilized as a comparison with the PIMs to test the influence of porosity (due to its lack of it). Figure 1 illustrates the molecular structures of the three polymers. By exploring this novel application, we aimed to investigate the potential of PIMs to enhance the functionality of graphene-based sensors for gas detection.

**Figure 1:**
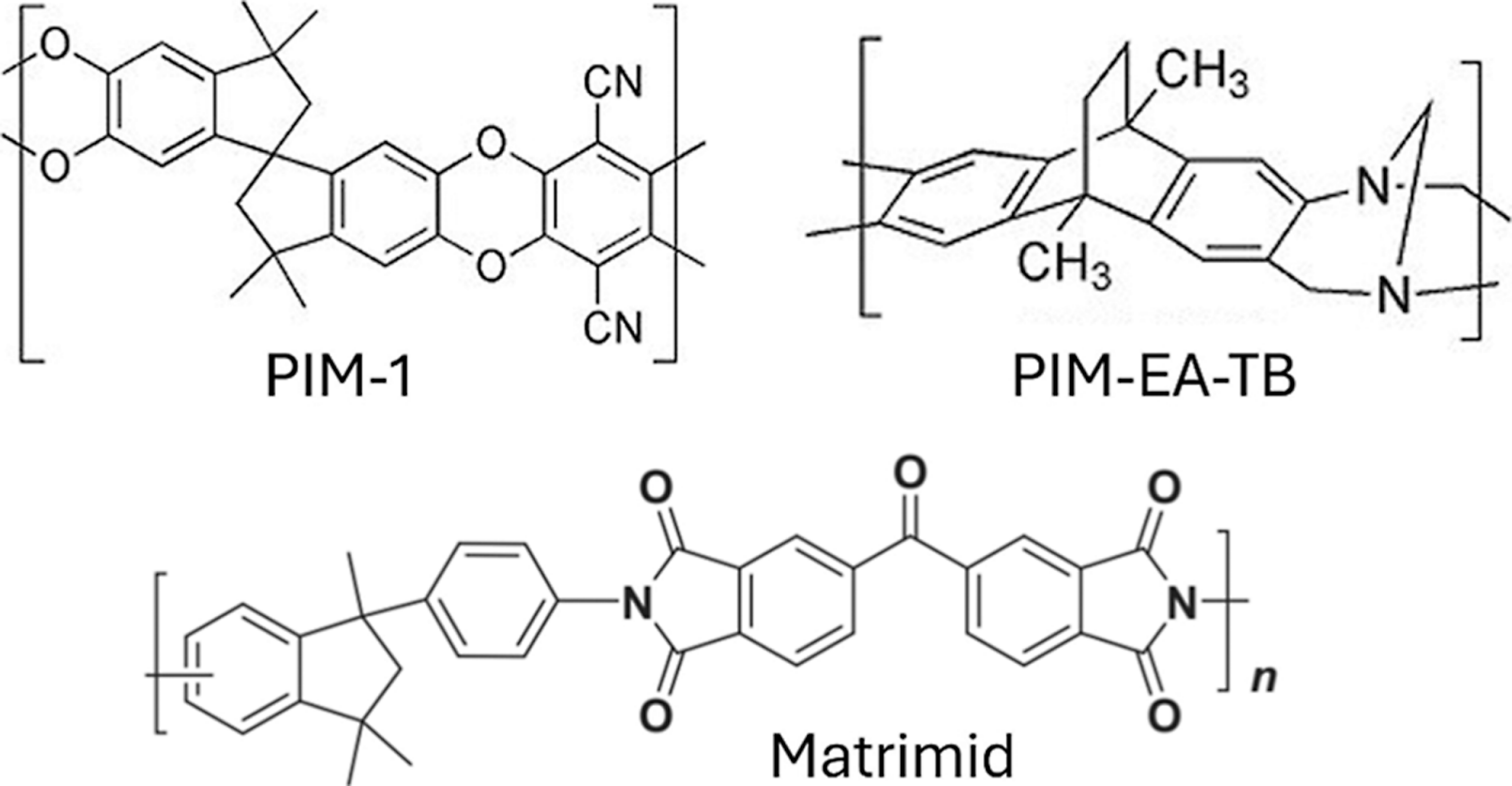
Molecular structure of the three polymers used in this study: PIM-1, PIM-EA-TB, and Matrimid.

## Experimental Section

### Materials

Chemical vapor deposited (CVD) monolayer graphene on 90 nm thermal oxide SiO_2_-381 μm Si wafers (⟨100⟩ orientation, 1–10 Ω cm resistivity and P-type/Bor) and monolayer graphene on 90 nm thermal oxide SiO_2_/381 μm Si wafers (⟨100⟩ orientation, 1–10 Ω cm resistivity and P-type/Bor) was supplied by Graphenea (Spain). Microposit LOR 3A Photoresist and Microposit S1805 G2 positive photoresist were supplied by DOW Electronics Materials (USA). TechniStrip NI555, 25% tetramethylammonium hydroxide (TMAH) etchant, AZ nLOF 2070 photoresist, and TI prime were supplied by MicroChemicals GmbH (Germany). Microposit MF-CD-26 Developer and Microposit 1165 Remover were supplied by A-Gas Electronic Materials (UK). Chromium and palladium PVD targets were supplied by Kurt J. Lesker Company Ltd. (UK). Trimethylaluminum (TMA) precursor was supplied by Pegasus Chemicals (UK). Type II DI water with the ASTM D1193 standard and a resistance of 18 MΩ.cm was produced using a Merck Millipore Elix 3 water purification system (Merck, Germany). Process gases were supplied by BOC Limited (UK); the concentrations employed were limited to BOC’s available products and the capabilities of the gas sensing system equipment. Chloroform was supplied by Fisher Scientific UK Ltd. (UK). Matrimid was supplied by Huntsman Corporation (UK). PIM-1 and PIM-EA-TB were synthesized by Dr Mariolino Carta’s research group (Swansea University, UK).

### Device Fabrication

**Figure 2:**
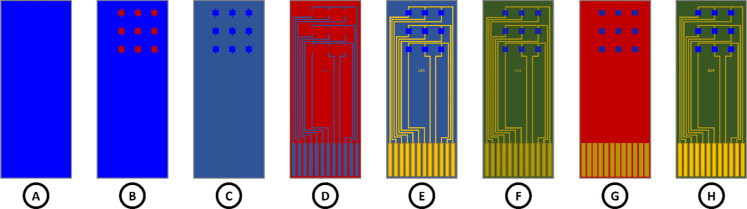
3 ×3 graphene pixel array device fabrication schematic. (A) CVD monolayer graphene on the Si/SiO_2_ substrate. (B) Application of a photoresist etch mask. (C) After O_2_ plasma etching and photomask removal. (D) Coating and patterning photoresist for metal electrodes. (E) Metal deposition and lift-off. (F) Deposition of the Al_2_O_3_ passivation layer using the MVD technique. (G) Coating and patterning photoresist for selective etching to expose graphene and metal contacts. (H) Al_2_O_3_ is etched, and photomask removed–finished sensor.

Fabrication was performed used an established process within the research group.^29^ Graphene sensors were fabricated using CVD single-layer graphene-on-90 nm SiO_2_/381 μm Si (1–10 Ω cm resistivity, p-type/Bor doping and ⟨100⟩ orientation), supplied by Graphenea (Figure 2A). To improve graphene-to-substrate adhesion prior to photolithography, wafers were annealed at 550 °C for 10 min using a Jiplec RTA system. Following annealing, wafers were coated in a bilayer photoresist composed of LOR 3A and S1805 to pattern the graphene pixels (Figure 2B). The coating, exposure, development, and removal processes were the same as those in our previous work. Graphene wafers were etched with O_2_ plasma using a Quorum Emitech K1050X RF Plasma Asher for 5 min, the photoresist mask protecting the areas where the graphene pixels would reside, while the excess graphene was etched away (Figure 2C). After the photoresist mask was removed, a second photolithography step was performed by using the same bilayer photoresist, this time patterning the metal contacts (Figure 2D). Wafers were loaded into a Kurt J. Lesker PVD75 system, and 30 nm Cr and 200 nm Pd were deposited onto them, connecting the graphene pixels to the 12 metal electrodes. Next, a lift-off procedure was used to remove the photoresist mask and excess metal, revealing the graphene sensors (Figure 2E). Finally, the devices were coated with a passivation layer, a 50 nm Al_2_O_3_ dielectric layer, deposited using an SPTS Technologies MVD300 system^29^ (Figure 2F). To create a window for the graphene pixels (400 × 400 μm) and metal electrodes, devices were coated and patterned using an AZ nLOF 2070 photoresist (Figure 2G). The substrate was immersed in 1.25% concentrated TMAH solution to etch the exposed Al_2_O_3_, exposing the graphene pixels and metal electrodes. After Al_2_O_3_ etching, the photoresist mask was removed with TechniStrip NI555 resist remover, revealing the final passivated 3 × 3 graphene pixel array devices (Figure 2H).

### Characterization

The spin-coated polymer films were characterized by using atomic force microscopy and ellipsometry. The methodologies for each technique are detailed below.

The surface topography of the spin-coated polymer films on 90 nm SiO_2_/381 Si wafers was characterized by using a Bruker Dimension Icon XR scanning probe microscope (Bruker Corporation, USA). The measurements were performed with a SCANASYST-AIR tip, featuring a resonant frequency of 70 kHz, a spring constant of 0.4 N/m, and a tip radius of 2 nm. The system operated in tapping mode at a line rate of 1.00 Hz, with a scan size of 250 × 250 nm.

Variable angle spectroscopic ellipsometry was performed using an M-2000 ellipsometer (J.A. Woollam, U.S.A.) to estimate the thickness of the spin-coated polymer films. Three angle scans were taken at 65°, 70°, and 75° based on the Brewster angle of the substrate (silicon). CompleteEASE software was used, and a Cauchy film optical model was employed to fit the data.

### Gas Sensing System

**Figure 3:**
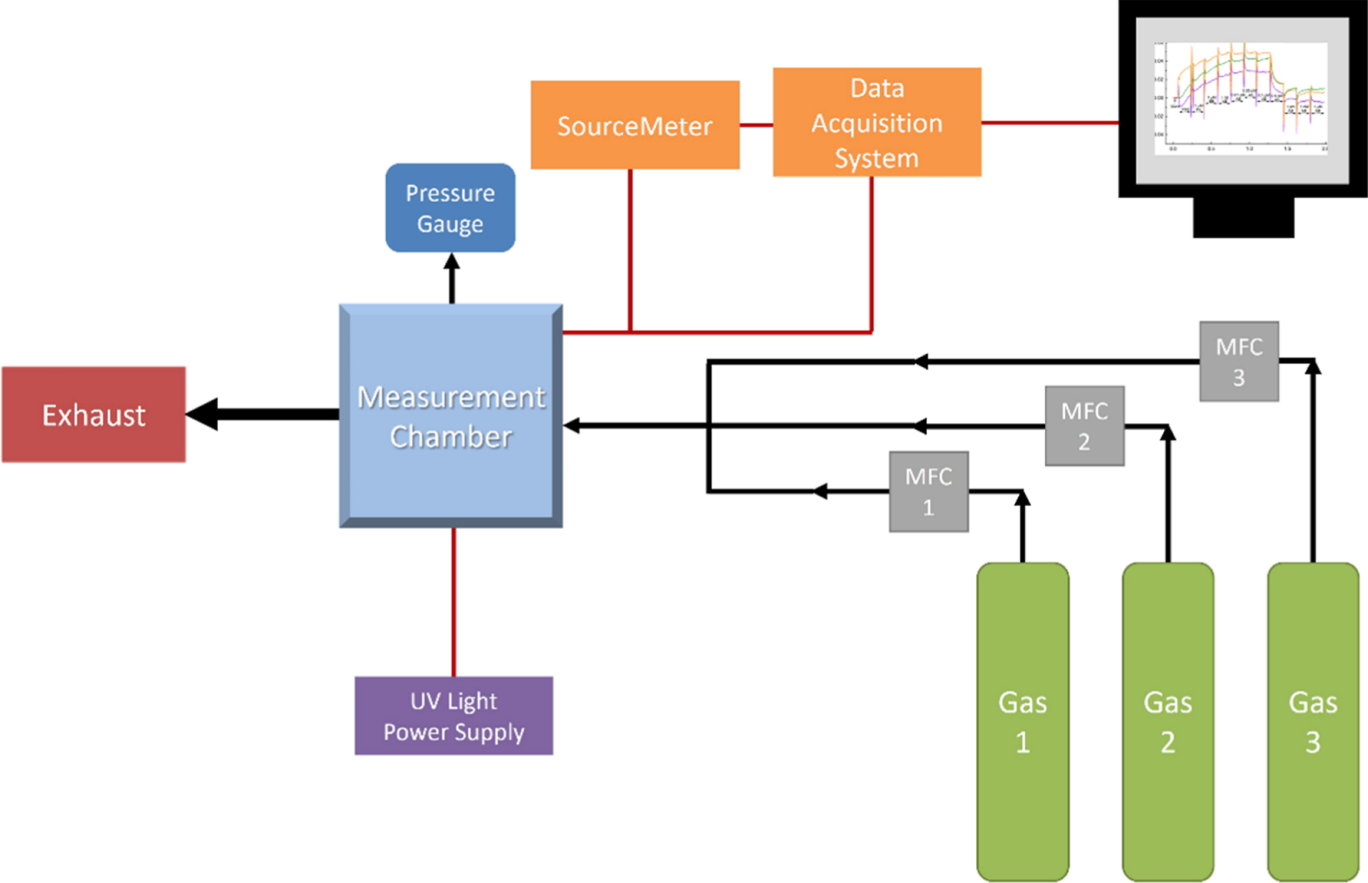
Schematic of the gas sensing system.

A custom-made gas sensing system created by the research group was used to test the sensing performance of the graphene devices. The system is composed of three mass flow controllers, a measurement chamber, electrical feedthroughs, exhaust/vacuum inlet, pressure gauge, and electrical measurement equipment (Figure 3).

### Electrical Measurements

**Figure 4:**
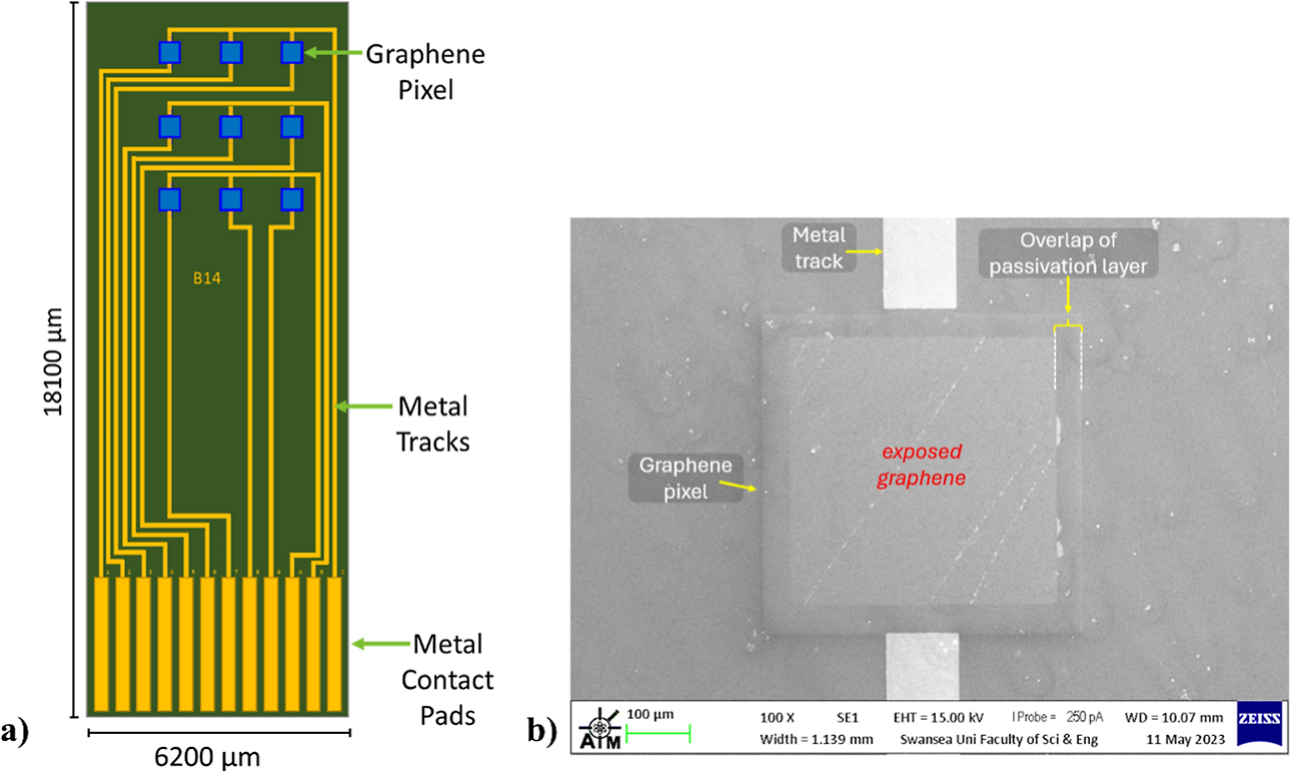
(a) Schematic of the 3 × 3 graphene array sensor. (b) SEM image of a graphene pixel on a 3 × 3 graphene pixel array device.

Real-time resistance measurements were performed using 3 × 3 graphene array sensors, consisting of nine CVD graphene pixels on a Si/SiO_2_ substrate (Figure 4). Pixels are measured simultaneously by sliding the sensors into a custom-made USB C PCB connector (purchased from Newbury Electronics, Newbury, UK) to provide an electrical connection between the metal contact pads and measurement equipment. An image of the connector can be found in Supporting Information Figure S1. Measurements were carried out under ambient conditions (temperature of 20 °C and normal atmospheric pressure). In the employed voltage-fixed regime, a voltage of 1 V was applied across the graphene pixels by using a Keithley 2602A SourceMeter (Tektronix, USA). The Keithley 6510 Data Acquisition/Multimeter System (Tektronix, USA), paired with the SourceMeter, allows for simultaneous resistance measurements of graphene pixels on the device. Following calculation of device resistance, the data are presented as Δ*R*/*R*_0_, where Δ*R* = *R*_g_ – *R*_0_ and *R*_0_ is the initial resistance of the device in ambient conditions.

### Graphene Surface Modification Process

For graphene surface modification, each of three 3 × 3 graphene pixel array devices were coated with one of the PIMs or Matrimid via spin-coating. Polymer solutions were prepared by dissolving each polymer powder in chloroform with a concentration of 1 mg/mL. Spin coating was performed for 30 s at 2500 rpm by using an L2001A3 Ossila spin coater with a custom-made chuck. During the spin-coating process, the metal contacts at the bottom of the devices were temporarily covered with a Parafilm to prevent them from being coated. This ensured that the surface modification was applied only to the desired areas of the sensor arrays.

## Results and Discussion

### Atomic Force Microscopy

**Figure 5:**
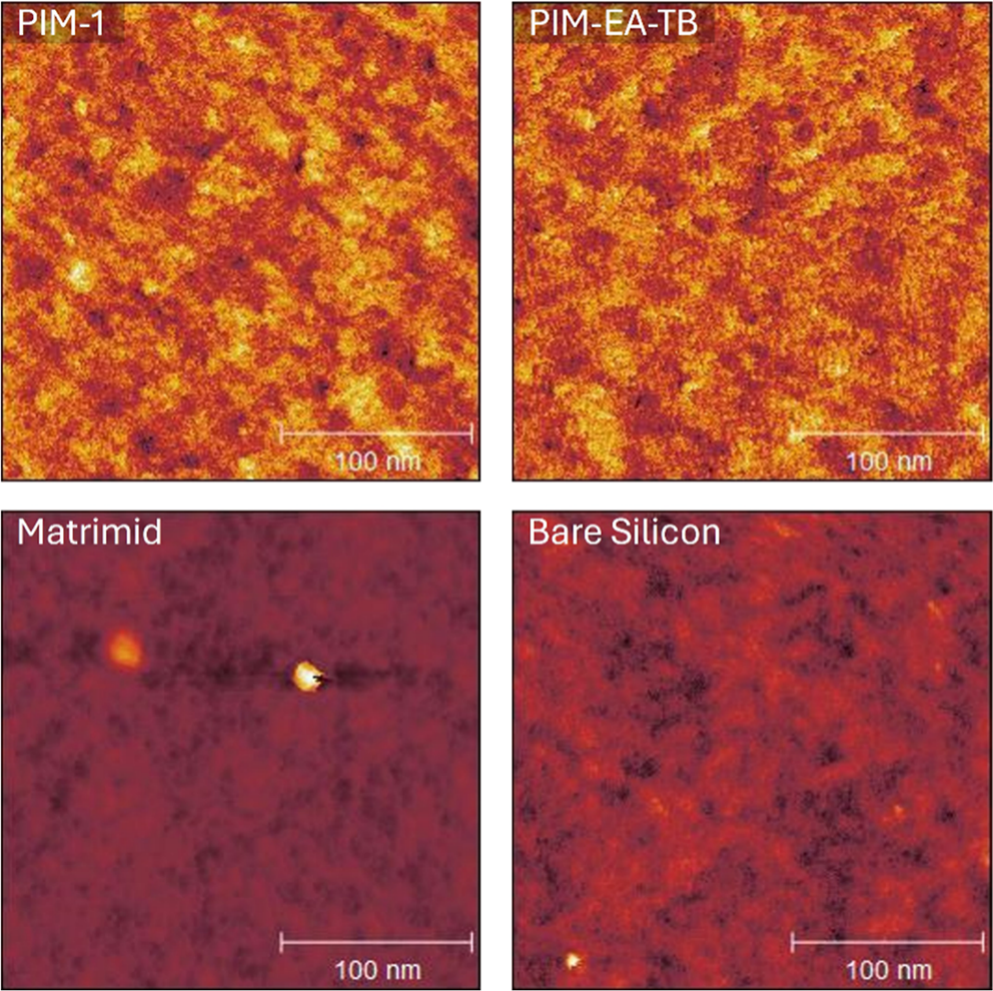
AFM images for a 250 nm spot size of PIM-1, PIM-EA-TB, Matrimid and a bare silicon wafer.

The surface topography of the spin-coated polymer films showed clear changes compared to that of the bare silicon wafer (Figure 5). The microstructures of PIM-1 and PIM-EA-TB are distinctly visible, revealing a distinctively rough surface texture. While the Matrimid polymer exhibits less pronounced surface features, which is expected as it is a denser and nonporous polymer, it shows an increase in surface roughness, with an RMS roughness of 0.213 nm compared to 0.112 nm for the bare silicon wafer. PIM-1 and PIM-EA-TB, as expected from their high porosity, exhibit even greater increases in surface roughness, with RMS roughness values of 0.285 and 0.293 nm, respectively.

These AFM measurements were performed primarily to understand the surface topography (particularly since these polymers are normally cast, not spin coated) and to verify surface modification. The increased roughness, indeed, is an indication of the successful surface modification, which was expected due to the nature of the polymers. The observed changes in surface roughness and topography serve as a valuable reference for future studies as a detailed investigation into how these characteristics influence gas sensing performance was beyond the scope of this work.

### Ellipsometry

The average film thicknesses measured for PIM-1, PIM-EA-TB, and Matrimid were found to be 10.56, 11.45, and 8.55 nm, respectively, with minimal variation across each spin-coated film. Although determining thickness is not deemed as critical to sensing performance at this stage, this characterization was conducted to establish a baseline for the polymer films given that these materials are often cast rather than spin coated. By quantifying the thickness, these data can serve as a reference should further optimization be needed. Future studies could explore whether varying film thicknesses impact sensor performance, such as sensitivity or selectivity, by affecting the dynamics of interaction between the gas molecules and the polymers.

### Gas Sensing Performance

This investigation reports on the sensor response, selectivity, sensitivity, and response and recovery times of the polymer-graphene hybrid sensors.

Real-time resistance measurements were performed in the gas sensing system on PIM-1-, PIM-EA-TB-, Matrimid-modified and bare graphene 3 × 3 graphene pixel array sensors. The devices were placed in the gas sensing chamber and exposed to 5 ppm of nitrogen dioxide (NO_2_). Selectivity was evaluated by exposing the sensors to individual gases, including 5 ppm ammonia (NH_3_), 5 ppm nitric oxide (NO), 100 ppm methane (CH_4_), and 450 ppm carbon dioxide (CO_2_), and measuring each respective response. All experiments commenced with a 10 min exposure to 20% oxygen/nitrogen gas (air) to establish a baseline, followed by a 10 min exposure to the target gas, and concluded with a 10 min exposure to air. The flow rate for all gas exposures was maintained at 1000 mL/min, and experiments were performed at room temperature (20 °C) and atmospheric pressure. The humidity of the sensing chamber was kept consistent throughout all experiments by flowing dry air into the chamber for 20 min to reduce the humidity, prior to conducting any sensing experiments. The sensor recovery was achieved through vacuum-assisted UV treatment using a 265 nm, 25 mW UV LED for a fixed duration of 8 min. This process ensured complete desorption of all gases prior to subsequent exposure to new gases.

**Figure 6:**
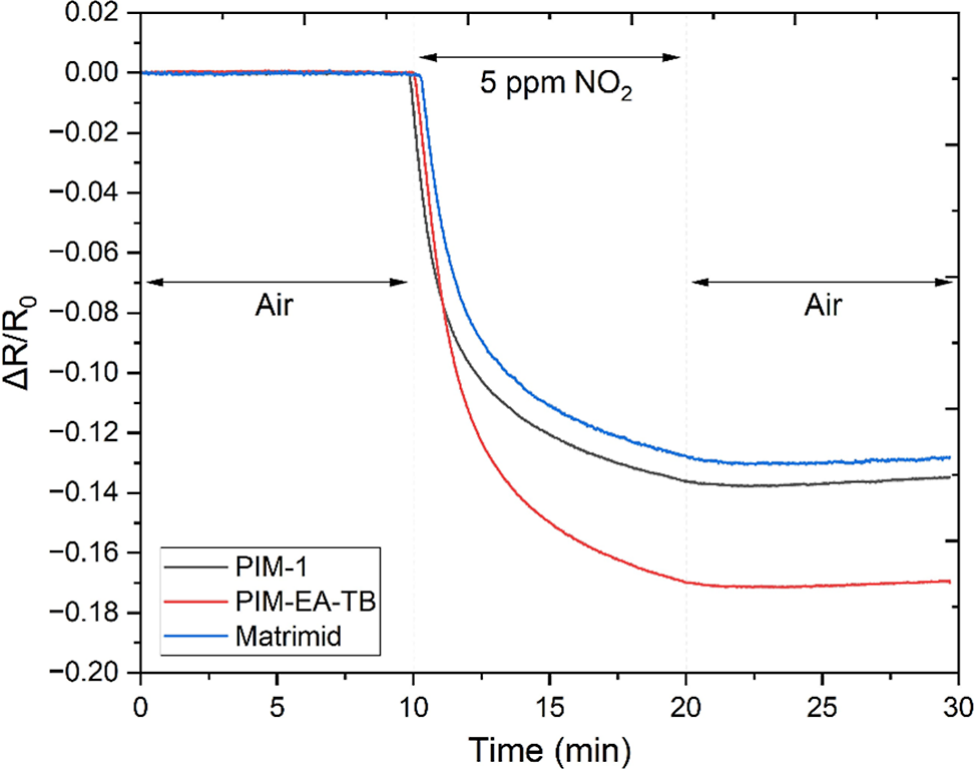
Real-time resistance measurements of three 3 × 3 graphene pixel array sensors coated with PIM-1, PIM-EA-TB, and Matrimid exposed to 5 ppm nitrogen dioxide expressed as ΔR/*R*_0_ (normalized resistance).

Figure 6 displays the real-time gas sensing response of the three polymer-coated sensors exposed to 5 ppm of NO_2_. The responses for each pixel on a device were averaged and presented as Δ*R*/*R*_0_ (normalized resistance), where Δ*R* = R_device_ – *R*_0_ and *R*_0_ is the resistance of the device in air. This normalization allows for easier comparison between the different sensors as the initial resistance may vary.

Upon exposure to NO_2_, there was a sharp decrease in the resistance of the sensor arrays, indicating a strong sensor response. However, subsequent exposure to air did not restore the sensors to their initial resistance values, suggesting incomplete recovery after NO_2_ exposure. The sensing mechanism of this device is attributed to the doped state of graphene and the characteristics of the target gas. Nitrogen dioxide, an electron-withdrawing gas, removes electrons from p-type graphene,^30,31^ increasing hole mobility and reducing resistance.^32^ While air exposure alone does not fully restore the sensor to its original baseline, subsequent exposure to UV light significantly increases resistance, rapidly returning the sensor to its baseline—and beyond—within 8 min by removing additional air adsorbates (O_2_)—also an electron withdrawing gas—from the surface. As adsorption of oxygen molecules to the surface would inherently decrease the resistance of p-type graphene, the removal of these molecules would cause the resistance to increase.^30,33^

The proposed mechanism for the desorption of molecules from the graphene surface is photodesorption. UV light provides the energy required to excite surface-bound species, facilitating their desorption from the surface. The vacuum further enhances this process by ensuring the efficient evacuation of desorbed species from the sensing chamber, thereby preventing readsorption. The following reaction mechanism^34,35^ illustrates this process
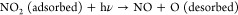
1

A real-time resistance response to nitrogen dioxide exposure, followed by UV-assisted recovery, is provided in the Supporting Information (Figure S2).

**Figure 7:**
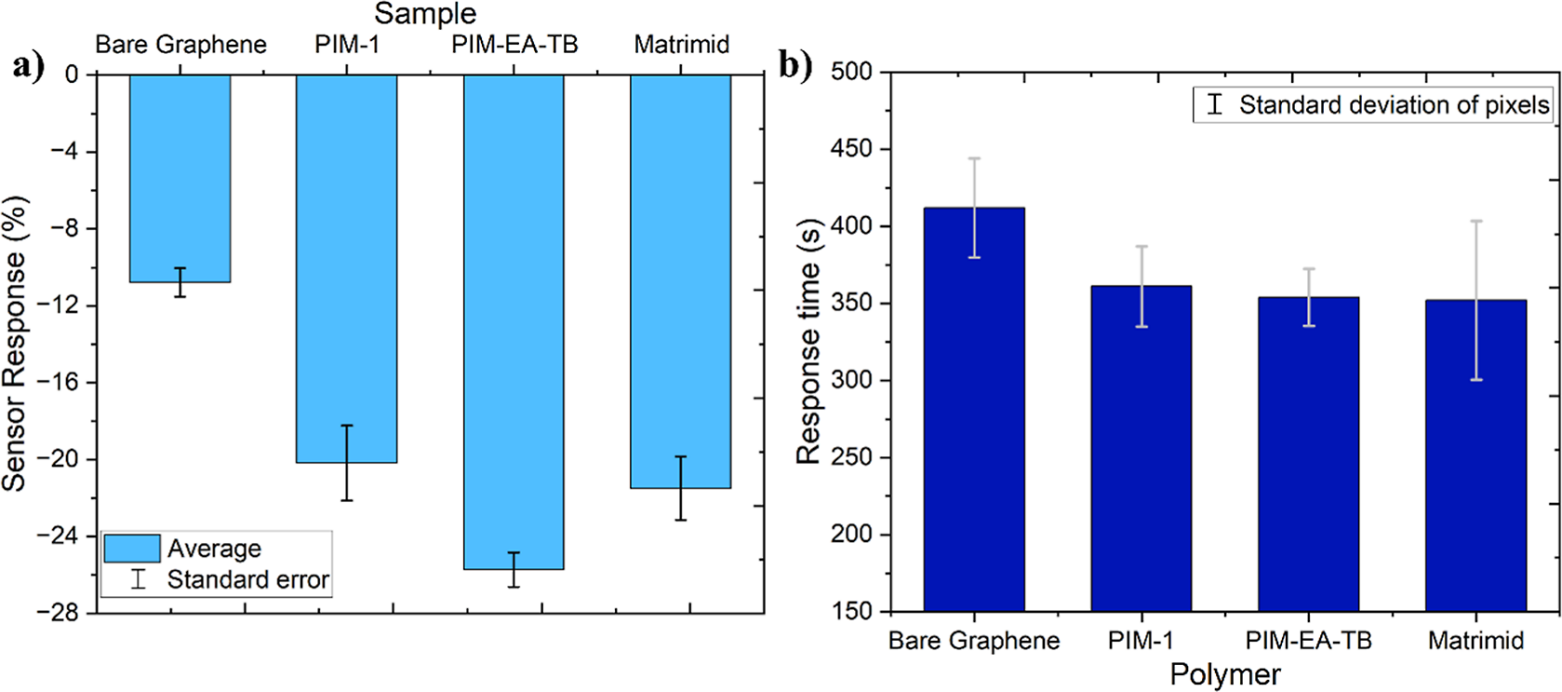
Bar chart displaying the (a) average sensor response (%) and (b) response time (s) of polymer-coated 3 × 3 graphene pixel arrays sensors exposed to 5 ppm of NO_2_ compared to the response of a bare graphene sensor.

The sensor response (Figure 7a), often expressed as a percentage of the relative response, is calculated by taking the difference between the maximum resistance in the presence of the target gas (*R*_g_) and the resistance in its absence (*R*_i_), divided by *R*_i_(36)
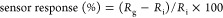


A negative sensor response indicates a decrease in the sensor’s electrical resistance upon exposure to the target gas, suggesting that the p-type graphene has interacted with an electron-withdrawing gas. Furthermore, the response times, defined as “the time required to reach 90% of the total change in electrical resistance in the presence of the target gas”,^35,37^ were calculated and shown in Figure 7b.

Figure 7 shows the average sensor response and response time of three surface-modified sensors, as well as a bare graphene sensor, when exposed to 5 ppm of NO_2_. The sensors coated with the polymers demonstrated significantly better performance than the bare graphene sensor, exhibiting both an increased sensor response and a shorter response time.

The bare graphene sensor, when exposed to 5 ppm of NO_2_, showed a response of −10.8% with a response time of 412 s. However, surface modification using PIM-1, PIM-EA-TB, and Matrimid notably improved the performance. The average sensor response for these modified sensors were −20.2%, −25.7%, and −21.5% respectively, while their response times decreased by approximately 56 s compared to the bare graphene sensor. The enhanced sensor performance highlights the potential of the modified sensors for detecting low concentrations of NO_2_. Moreover, the spin-coating modification method is quick, simple, and efficient, offering a practical and effective means of achieving significant improvements in sensor performance.

The increase of performance in the PIM-coated sensors can be attributed to the kinetic diameter of the nitrogen dioxide molecule, which is 3.3 Å^38,39^—identical to that of the carbon dioxide (CO_2_) molecule for which PIMs are known to be size selective. PIMs, in fact, demonstrate enhanced diffusivity-selectivity toward molecules with small kinetic diameters.^22^ Compared to nitrogen (N_2_) and oxygen (O_2_), with kinetic diameters of 3.64 and 3.46 Å, respectively, the smaller NO_2_ allowed to pass through the membrane more easily. Thus, the molecular sieving effect provided by PIMs seems to be enhancing the sensor’s performance by preferentially allowing smaller molecules to diffuse through, increasing the sensitivity to NO_2_.

To investigate how different concentrations of nitrogen dioxide affect sensor response, the three polymer-modified 3 × 3 graphene pixel array sensors were tested with a range of NO_2_ concentrations: 1, 2.5, 5, 10, 25, and 50 ppm. To enable gas dilution, the gas lines were merged prior to entering the sensing chamber, allowing thorough mixing before exposure to the sensors. The flow rate of the target gas was reduced, while air was added to achieve a total flow rate of 1000 mL/min.

**Figure 8:**
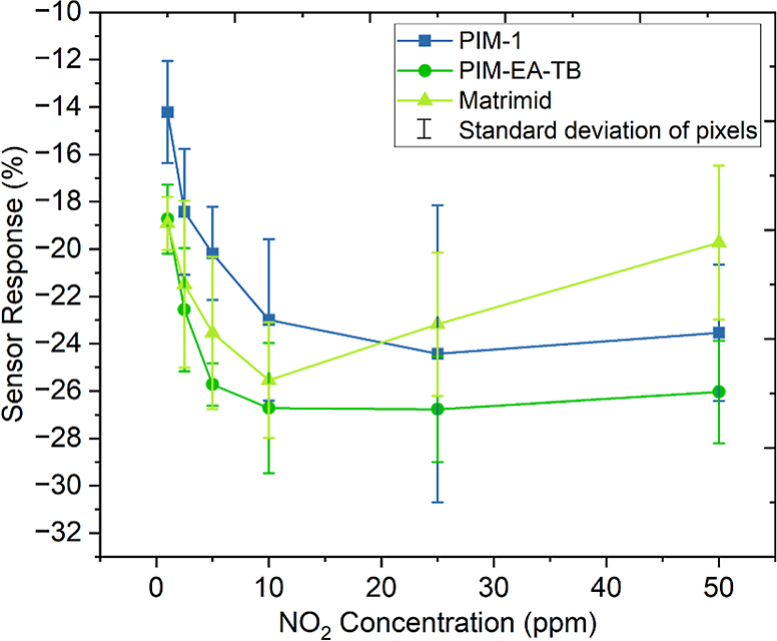
Sensor response (%) versus parts per million (ppm) NO_2_ concentration at various concentrations ranging from 1 to 50 ppm for PIM-1-, PIM-EA-TB-, and Matrimid-coated 3 × 3 graphene pixel array sensors.

Figure 8 displays the response to various NO_2_ concentrations for each polymer-coated sensor. All three devices exhibit an improved response with an increased NO_2_ concentration up to 25 ppm, beyond which sensor signals appear to approach saturation. At 25 ppm, the Matrimid-coated sensor shows a significant decrease in response, the PIM-EA-TB sensor shows a slight decrease, and the PIM-1 sensor decreases similarly at 50 ppm. Given the thin film thickness of the polymer membranes (∼9–11 nm), this trend suggests that the polymers are likely saturating with NO_2_ at higher concentrations. Additionally, at these elevated concentrations, the vacuum-assisted UV treatment may not be effectively removing all of the NO_2_ from the polymer membranes and graphene surface. This could result in a diminished sensor response during subsequent exposures.

Although the recovery period for each repetition was fixed at 8 min to ensure complete sensor recovery, the actual recovery time for exposure to 5 ppm of NO_2_ was still estimated. This was performed by identifying the point within the 8 min recovery period where the baseline was recovered. The normalized resistance (Δ*R*/*R*_0_) for each graphene pixel was averaged over three repetitions from which the recovery time was estimated. The results show recovery times of 114 s for the PIM-1 sensor, 149 s for the PIM-EA-TB sensor, and 153 s for the Matrimid-coated sensor. These results proved to be excellent, especially when compared to other NO_2_ graphene-based sensors reported in the literature.^40−45^ A detailed comparison of the gas sensing performance of these sensors is provided in the following section.

The selectivities of the PIM-1-, PIM-EA-TB-, and Matrimid-coated sensors were evaluated by exposing them independently to various gases and measuring the sensor response: 5 ppm ammonia (NH_3_), 5 ppm nitric oxide (NO), 100 ppm methane (CH_4_), and 450 ppm carbon dioxide (CO_2_).

**Figure 9:**
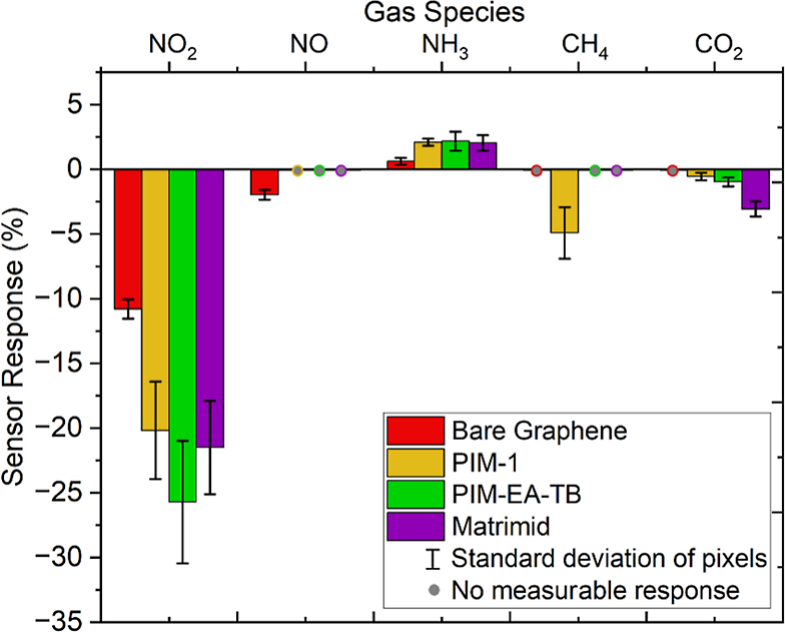
Bar chart comparing the selectivity of bare graphene, PIM-1-, PIM-EA-TB-, and Matrimid-coated 3 × 3 graphene pixel array sensors exposed to various gases: 5 ppm nitrogen dioxide (NO_2_), 5 ppm ammonia (NH_3_), 5 ppm nitric oxide (NO), 100 ppm methane (CH_4_), and 450 ppm carbon dioxide (CO_2_).

Figure 9 compares the selectivity of polymer-coated sensors to various gases against that of a bare graphene sensor. Our devices exhibited the highest selectivity toward NO_2_. The polymer-coated sensors showed no measurable response to NO and only small responses to NH_3_ (ranging from 2.0% to 2.2%), although they were still higher than those of bare graphene (0.63%). This suggests that the polymers do not significantly enhance the selectivity for NH_3_ or NO. While the responses to CH_4_ and CO_2_ were relatively small, PIM-1 exhibited a response to CH_4_ (−4.9%), and all three polymer-coated sensors responded to CO_2_, with responses ranging from −0.55% to −3.1%. In contrast, bare graphene showed no response to either of these gases. This indicates that the polymer membranes selectively allow these gases to pass through more easily than O_2_ and N_2_, thereby triggering a response from the underlying bare graphene.

The sensing performance of the polymer-coated sensors was evaluated in comparison to bare graphene and similar sensors documented in the literature. Table 1 compares the sensor response, recovery time, and percentage recovery for the sensors in this study with other room temperature NO_2_ sensors using graphene materials.^40−45^

**Table 1:** Comparison of Graphene-Based NO_2_ Sensors Using Different Sensing Materials

sensing materials	NO_2_ response	recovery time (s)	recovery (%)	refs
CVD graphene/PIM-1	20% at 5 ppm	114	100	this work
CVD graphene/PIM-EA-TB	26% at 5 ppm	149	100	this work
CVD graphene/Matrimid	22% at 5 ppm	153	100	this work
CVD graphene	11% at 5 ppm	93	100	this work
4-aminoquinoline-rGO	74% at 10 ppm	3300	80	(40)
CVD graphene	32% at 200 ppm	>1440	did not recover in time allowed	(41)
CVD graphene	26% at 100 ppm	800	not reported	(34)
rGO-coated nanofibrous mesh fabrics	14% at 1 ppm	10000	not reported	(42)
ME graphene	5% at 1.5 ppm	1200	not reported	(43)
CVD graphene	20% at 1 ppm	900	97	(44)
CVD graphene-AlGaN/GaN	19% at 5 ppm	>600	full recovery observed after overnight	(45)
SnO_2_-rGO hybrid	2% at 1 ppm	100	not reported	(46)

A comparison of the polymer-coated sensors with other sensors demonstrates their potential for highly sensitive, selective, and rapid recovery devices. While some studies have reported larger responses,^40,44^ often at higher concentrations, the recovery times achieved in this work (ranging from 67 to 153 s) and the complete 100% recovery of the devices are superior. Although the metal oxide sensor (SnO_2_-rGO hybrid) demonstrates a slightly quicker recovery time compared to the polymer-coated sensors, its response magnitude to NO_2_ is significantly lower. This suggests that the devices reported in this work exhibit a higher level of sensitivity. Notably, the PIM-coated sensors also show impressive selectivity toward NO_2_, further highlighting their superior performance in detecting this gas. Besides, the limit of detection (LOD) and limit of quantitation (LOQ) were estimated for each of the polymer-coated sensors and are shown in Table 2. The theoretical detection limits were calculated by using the slope from the linear portion of the calibration curve (Figure S3). The low LOD and LOQ values, in the range of a few parts per billion (ppb), further suggest that these sensors have the potential to be highly sensitive NO_2_ detectors.

**Table 2:** Estimated LOD and LOQ for Each Polymer-Coated 3 × 3 Graphene Pixel Array Sensor

polymer	LOD (ppb)	LOQ (ppb)
PIM-1	1.0	3.4
PIM-EA-TB	0.7 (700 ppt)	2.3
Matrimid	1.0	3.3

## Conclusions

In this study, we fabricated and evaluated the gas sensing performance of 3 × 3 graphene pixel array sensors coated with PIM-1, PIM-EA-TB, and Matrimid. Matrimid, a commercial polyimide thermoplastic commonly used in gas separation, was employed as a benchmark for comparison. Both the PIMs and Matrimid showed excellent selectivity for NO_2_, with sensor responses 9–15% higher than those of bare graphene, alongside faster response times. Compared to similar systems reported in the literature, these polymer-coated sensors not only exhibit stronger responses but also demonstrate faster recovery times, outperforming existing devices. The estimated LOD and LOQ were in the low ppb range, making these sensors highly relevant for real-world applications where even trace levels of NO_2_ pose significant health risks, particularly to respiratory systems. Overall, the PIM-graphene hybrid sensors demonstrate great promise as highly sensitive and selective NO_2_ detectors, and when combined with the straightforward surface modification process, they offer the potential for developing accurate, reliable, and cost-effective sensors for real-time environmental monitoring.

## Supporting Information Available

The USB-C PCB connector employed, real-time resistance measurements recorded during UV recovery, and the linear fit applied to the linear portion of the calibration curve (PDF)

## Abbreviation

PIM: polymer of intrinsic microporosity
