# Supplementary material for: Enhanced Nitrogen Dioxide Detection Using Resistive Graphene-Based Electronic Sensors Modified with Polymers of Intrinsic Microporosity
Source: ACS Sens. 2025 Feb 17;10(2):1378–86. doi: 10.1021/acssensors.4c03291 (PMC11877633; doi:10.1021/acssensors.4c03291)
Supplement: Supplementary file 1 — se4c03291_si_001.pdf [file se4c03291_si_001.pdf]

Enhanced Nitrogen Dioxide Detection Using Resistive Graphene-based Electronic Sensors Modified with Polymers of Intrinsic Microporosity

Danielle M. Goodwin †, Mariolino Carta‡, Muhammad Munem Ali†, Daniel Gillard† and Owen J. Guy* †, ‡*

†Centre for Integrative Semiconductor Materials (CISM), Faculty of Science and Engineering, Swansea University - Bay Campus, Fabian Way, Swansea SA1 8EN, UK

‡Department of Chemistry, College of Science, Swansea University - Singleton Campus, Swansea SA2 8PP, UK

Corresponding authors: 1914696@swansea.ac.uk; O.J.Guy@Swansea.ac.uk

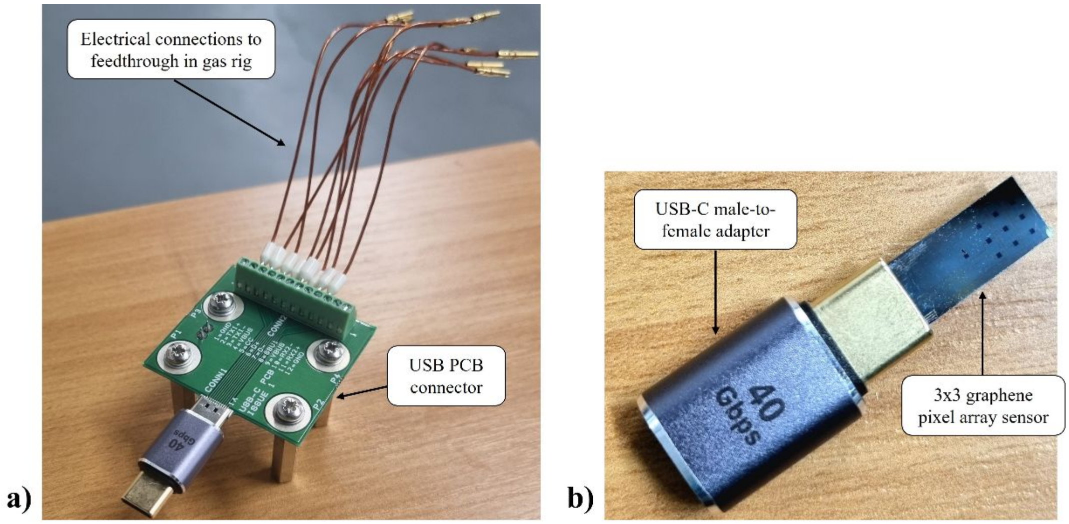

Figure S1. (a) USB-C PCB connector with USB-C male to female adapter and wire connections for D-sub electrical feedthrough and (b) USB-C male to USB-C female adapter with 3x3 graphene array sensor.

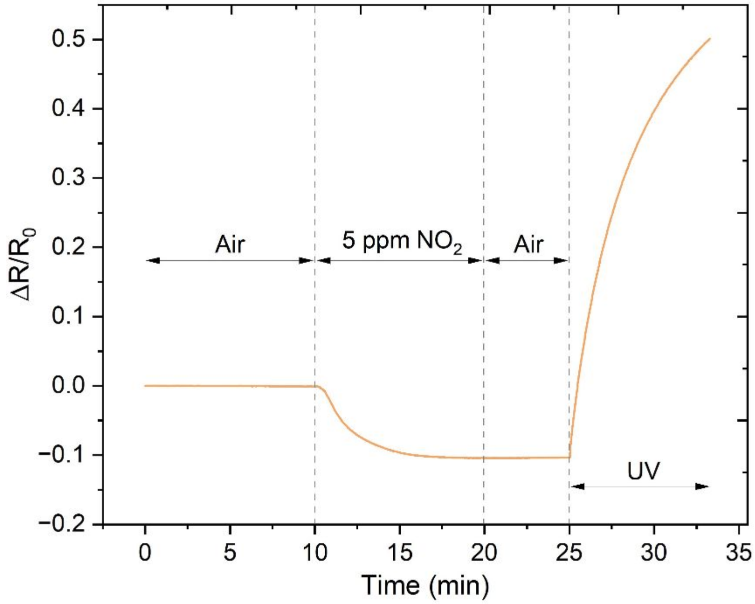

Figure S2. Real-time resistance measurements from a graphene pixel array sensor demonstrating a 10-minute exposure to 5 ppm NO_2 followed by an 8-minute vacuum assisted exposure to UV light.

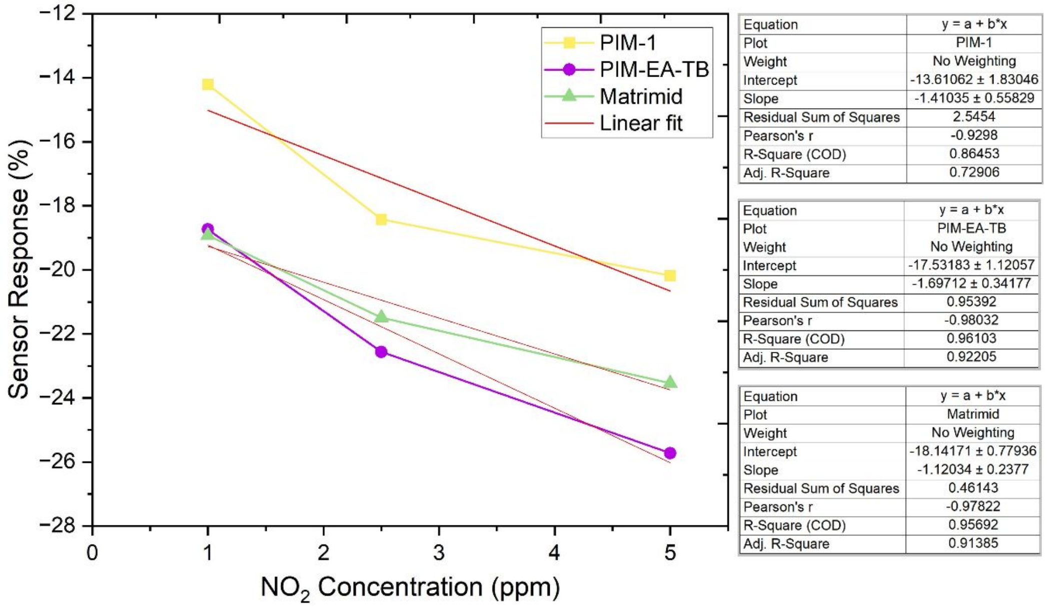

Figure S3. Linear fit for the linear portion of calibration curve obtained from the concentration range 1 ppm to 5 ppm using the polymer-functionalized 3x3 graphene pixel array sensors. Origin linear fitting software was used to perform the fit.
